# The Influence of Maternal KIR Haplotype on the Reproductive Outcomes after Single Embryo Transfer in IVF Cycles in Patients with Recurrent Pregnancy Loss and Implantation Failure—A Single Center Experience

**DOI:** 10.3390/jcm12051905

**Published:** 2023-02-28

**Authors:** Radu Maftei, Bogdan Doroftei, Radu Popa, Valeriu Harabor, Ana-Maria Adam, Cristina Popa, AnaMaria Harabor, Gigi Adam, Aurel Nechita, Ingrid-Andrada Vasilache, Elena Mihalceanu, Anca Bivoleanu, Gabriela Lunguleac, Ana-Maria Cretu, Teodora Armeanu, Roxana Diaconu, Petru Cianga

**Affiliations:** 1 Department of Mother and Child Care, “Grigore T. Popa” University of Medicine and Pharmacy, 700115 Iasi, Romania; 2 Department of Vascular Surgery, University of Medicine and Pharmacy “Grigore T. Popa”, 700111 Iasi, Romania; 3 Clinical and Surgical Department, Faculty of Medicine and Pharmacy, ‘Dunarea de Jos’ University, 800216 Galati, Romania; 4 Discipline of Oral Medicine, ‘Grigore T. Popa’ University of Medicine and Pharmacy, 700115 Iasi, Romania; 5 Department of Pharmaceutical Sciences, Faculty of Medicine and Pharmacy, ‘Dunarea de Jos’ University, 800216 Galati, Romania; 6 Department of Immunology, ‘Grigore T. Popa’ University of Medicine and Pharmacy, 700115 Iasi, Romania

**Keywords:** recurrent pregnancy loss, recurrent implantation failure, Kir haplotype, HLA-C

## Abstract

(1) Background: Recurrent pregnancy loss (RPL) and recurrent implantation failure (RIF) have in common a deficient maternal adaptation to the semi-allogeneic fetus, in which killer immunoglobulin-like receptor (KIR) family expressed by natural killer (NK) cells play an important role. The aim of this study was to evaluate the influence of maternal KIR haplotype on the reproductive outcomes after single embryo transfer in IVF cycles in patients with RPL and RIF. (2) Methods: Patients with RIF and RPL who presented at Origyn Fertility Center from Iasi, Romania, were prospectively enrolled between January 2020 and December 2022. Clinical and paraclinical data was examined. Descriptive statistics and a conditional logistic regression model were used to analyze our data. (3) Results: Patients with a KIR AA haplotype had significantly more chances of miscarriage if they underwent an IVF procedure (aOR: 4.15, 95% CI: 1.39–6.50, *p* = 0.032) compared with those who spontaneously achieved a pregnancy. Moreover, it appeared that the same haplotype increased the chances of obtaining a pregnancy for patients who underwent an IVF procedure (aOR: 2.57, 95% CI: 0.85–6.75, *p* = 0.023). (4) Conclusions: Determination of KIR haplotype could be beneficial for patients with RPL or RIF in order to offer an individualized management.

## 1. Introduction

The definition of recurrent pregnancy loss (RPL) varies in the literature, but one of the most used definitions describes this disorder as the spontaneous loss of two or more pregnancies [1]. The prevalence of pregnancy loss is difficult to estimate since it depends on how early women discover their pregnancy, the study population, and the use of multiple criteria to diagnose this disorder. Recent reports indicate an average prevalence of recurrent pregnancy loss between 1% and 4% of all women who achieve pregnancy [2,3]. 

On the other hand, recurrent implantation failure (RIF) refers to cases in which women have had three failed in vitro fertilization (IVF) attempts with good quality embryos [4]. The definitions of RIF reported in the literature are often heterogeneous, and so, an accurate epidemiological profile of this disorder cannot be properly specified. However, one recent retrospective cohort study by Pirtea et al. involved 4429 women with an anatomically normal uterus, who underwent up to three consecutives frozen euploid single embryo transfers estimated an RIF prevalence of less than 5% [5].

Both RPL and RIF appear to have in common a deficient maternal adaptation to the semi-allogeneic fetus [6,7,8,9]. An important element in the pathophysiology of these illnesses is represented by the natural killer (NK) cells, and their interaction with various ligands [10]. Because of their ability to release cytokines and destroy target cells without prior sensitization, NK cells play a fundamental role in the innate immune response. Due to their cytotoxic nature, NK cells must be able to distinguish normal self-tissue in order to prevent self-destruction [11]. Since both maternal KIR and fetal HLA-C genes are extremely variable, communication among members of the killer immunoglobulin-like receptor (KIR) family produced by NK cells and trophoblast human leukocyte antigen-C (HLA-C) molecules seem to be of particular consequence in terms of allorecognition [12].

In any pregnancy, the maternal KIR genotype could be AA (mostly inhibitory KIRs), AB, or BB (mostly activating KIRs) [13]. Seven KIR genes make up the KIR AA haplotype (3 inhibitory, 3 framework and 1 nonfunctional activating genes) [14]. The KIR BB haplotype is unique because it contains a number of extra KIRs that are responsible for activation. The KIR locus has distinct centromeric (cen) and telomeric (tel) ends. A KIR AA haplotype is defined as cen-A and tel-A, while a KIR Bb haplotype is described as cenB/telB, cenA/telB, or cenB/telA [14]. HLA-C ligands for KIRs are classified into two types: HLA-C1 and HLA-C2. The inhibitory receptors KIR2DL2 (B haplotype) and KIR2DL3 (A haplotype) are ligands for the C1 group allotypes, while the activating KIR2DS1 receptors are ligands for the C2 group allotypes (B haplotype). C2 is a more potent ligand than C1 [15].

In recent years, several studies have outlined the association of various KIR polymorphisms with infertility, and pregnancy related disorders, but there is still great heterogeneity over the reporting of specific KIR haplotypes and their influence on the reproductive outcomes [16,17,18]. Moreover, a systematic review by Wong et al. assessed the influence of various immunotherapies on the live birth rate in women with RPL, and the authors concluded that paternal cell immunization, third-party donor cell immunization, trophoblast membrane infusion, and intravenous immunoglobulin did not have a significant influence over the evaluated outcome [19].

The aim of this study was to evaluate the influence of maternal KIR haplotype on the reproductive outcomes after single embryo transfer in IVF cycles in patients with recurrent pregnancy loss and implantation failure in a cohort of patients from Romania.

## 2. Materials and Methods

In this study, patients with RIF and RPL who presented for consultations at Origyn Fertility Center in Iasi, Romania, were prospectively enrolled between January 2020 and December 2022. The Institutional Ethics Committee of the University of Medicine and Pharmacy “Grigore T. Popa” granted ethical permission for this research (No. 143/18.03.2019). All individuals provided informed permission prior to inclusion in the research. All procedures were carried out in conformity with applicable rules and norms.

The inclusion criteria taken into consideration were: pregnant patients with RPL or RIF, age ≥18, with/without previous IVF procedure. We considered RPL as the spontaneous loss of two or more pregnancies [1], and RIF as three failed in vitro fertilization (IVF) attempts with good quality embryos [4]. For determining the embryo’s quality, we used the Istanbul consensus grading system for embryo assessment [20]. A good-quality embryo was considered to have a 4:1:1 grading, corresponding to a hatched blast, with a prominent inner cell mass (ICM), and the trophectoderm (TE) appearance of a cohesive epithelium. All embryos with this grade were further selected for transfer. Individuals with ectopic pregnancies, fetal intrauterine death, fetuses with chromosomal or anatomical impairments, missing health records, or an inability to give informed permission were excluded from the study.

A thorough fertility examination was performed on each woman and her companion. This involved taking a full medical history, performing a physical exam, testing for viruses (hepatitis B and C, and HIV), analyzing serum hormone levels (including thyroid-stimulating hormone [TSH], free thyroxin [fT4], anti-thyroid peroxidase antibodies [ATPO], anti-mullerian hormone [AMH], and 25-hydroxy vitamin D [25(OH)D]), analyzing spermogram, and performing a pelvic sonogram. The parental karyotype, thrombophilic diseases screen, anticardiolipin, anti-b-2-glycoprotein, and lupus anticoagulant tests were carried out.

Each participant had 5 mL of blood collected into tubes containing ethylenediaminetetraacetic acid (EDTA). The following KIRs were typed using polymerase chain reaction with sequence-specific primers (PCR/SSP): 2DL1, 2DL2, 2DL3, 2DL4, 2DL5, 2DS1, 2DS2, 2DS3, 2DS4, 2DS4N, 2DS5, 3DL1, 3DL2, 3DL3, 3DS1, 2DP1, and 3DP1. The genotype was considered as B if any of the genes 2DL2, 2DL5, 3DS1, 2DS1, 2DS2, 2DS3, and 2DS5 were present. If none of these were found, AA was regarded as the genotype.

PCR analysis was also used to examine the HLA-C genotypes. The HLA-C genes of all spouses or egg donors were examined and categorized as C1 HLA-C or C2 HLA-C. All HLA-C alleles in individuals were classified as C1 or C2 based on the amino acid present at position 80 of the HLA-C molecule (asparagine or lysine). KIRs attach to HLA-C molecules at this location, to the C1 or C2 CIR-binding epitope. The genetic testing for both KIR and HLA-C was performed at ‘Queen Mary’s Genetic Center’ in Bucharest, Romania.

We chose to use a theoretical score proposed by Alecsandru et al. [12] and defined the number of C2 alleles from both gamete providers, multiplied by the number of embryos transferred, minus the number of C2 alleles held by the mother for evaluation of excessive maternal HLA-C2 presence.

We included 108 patients out of the 123 patients initially evaluated, and we divided them into three subgroups: subgroup 1 (AA genotype, n = 31), subgroup 2 (BB genotype, n = 72), and subgroup 3 (AB genotype, n = 5). The following reproductive outcomes were assessed: the pregnancy rate, the miscarriage rates, and the live birth rates. These reproductive outcomes were further segregated depending on the mode of achieving a pregnancy (IVF or spontaneous).

In the first phase of the statistical analysis, as part of the descriptive statistics, each categorical data were assessed using chi-squared and Fisher’s exact tests, and were reported as percentages and frequencies, while each continuous data were evaluated using t-tests, and were displayed as means and standard deviations (SD).

Using an analysis of variance (ANOVA) followed by the Bonferroni post hoc test, the extent of statistical difference between the subgroups in terms of their biohumoral features was determined. STATA SE was used to conduct the statistical analysis (version 17, 2021, StataCorp LLC, College Station, TX, USA).

A conditional logistic regression (CLR) model was applied for the evaluation of associations between reproductive outcomes and KIR haplotypes and the adjusted odd ratios (aOR) with 95% Confidence Intervals (CI) were calculated for each variable of interest. The patient’s clinical characteristics were used as confounders in the regression analysis. A *p* value of less than 0.05 was considered statistically significant.

## 3. Results

A total of 108 pregnant patients with recurrent pregnancy loss or recurrent implantation failure were assessed and included in our prospective study. Table 1 presents their clinical features, which were categorized into the following groups: RPL (group 1, 30 patients), and RIF (group 2, 78 patients). The personal history of systemic lupus erythematosus (SLE)/antiphospholipid syndrome (APS) (*p* = 0.005), thrombophilia (*p* = 0.002), and thrombosis (*p* = 0.008) were significantly more prevalent in the group of patients with recurrent pregnancy loss. On the other hand, we could not find any significant difference between groups regarding their personal history of hypertension, renal disease, diabetes, endometriosis, autoimmune thyroiditis, rheumatoid arthritis, or autoimmune thrombocytopenic purpura (*p* > 0.05).

The paraclinical characteristics of the main groups are presented in Table 2. Our results indicated that ATPO serum levels were significantly higher in the RPL group compared to RIF group (140.32 ± 378.48 versus 50.89 ± 156.99 UI/mL, *p* = 0.005). Moreover, the vitamin D serum levels were significantly lower in the RPL group compared to RIF group (32.76 ± 21.98 versus 39.91 ± 20.13 ng/mL, *p* = 0.006). Although the prevalence of AA haplotypes was higher in the RPL group, and the prevalence of AB haplotype was higher in the RIF group, the difference was not statistically significant. The percentage of BB haplotype was the same for the examined groups (66.7%). Additionally, the HLA-C variants did not significantly differ among the evaluated groups.

Subgroup 1 (AA genotype, n = 22), subgroup 2 (BB genotype, n = 94), and subgroup 3 (AB genotype, n = 88) were subjected to further comparative analyses based on their biohumoral features (Table 3). The analysis of variance (ANOVA) followed by the Bonferroni post hoc test revealed that there was not a statistically significant difference between the exanimated categories with relation to their biohumoral parameters.

A comparison of the reproductive outcomes is presented in Table 4. Our results indicated that the miscarriage rate was significantly lower (6.9%, *p* = 0.018), and the live birth rate (87.5%) was significantly higher for BB haplotype compared to other haplotypes. On the other hand, no statistically significant difference could be determined between haplotypes regarding the pregnancy rate.

The reproductive outcomes were further comparatively analyzed based on the KIR genotype (Table 5). Patients with an AA haplotype had significantly more chances of miscarriage if they underwent an IVF procedure (aOR: 4.15, 95% CI: 1.39–6.50, *p* = 0.032) compared with those who spontaneously achieved a pregnancy. The chances of obtaining a pregnancy were also significantly higher for those patients with an AA haplotype who underwent an IVF procedure (aOR: 2.57, 95% CI: 0.85–6.75, *p* = 0.023), but at the same time, live birth odds did not significantly differ between the evaluated haplotypes.

## 4. Discussion

In this prospective study, we assessed the influence of maternal KIR haplotypes on reproductive outcomes after single embryo transfer in IVF cycles in patients with recurrent pregnancy loss and implantation failure. Our results indicated that the miscarriage rate was significantly lower (6.9%, *p* = 0.018) and the live birth rate (87.5%) was significantly higher for BB haplotype compared to other haplotypes. On the other hand, when we stratified the reproductive outcomes using the modality of achieving a pregnancy, patients with an AA haplotype had significantly more chances of a miscarriage if they underwent an IVF procedure (aOR: 4.15, 95% CI: 1.39–6.50, *p* = 0.032) compared with those who spontaneously achieved a pregnancy. Moreover, it appeared that the same haplotype increased the chances of obtaining a pregnancy for patients who underwent an IVF procedure (aOR: 2.57, 95% CI: 0.85–6.75, *p* = 0.023).

These findings are complementary to those previously published in the literature. For example, in a retrospective study by Alecsandru et al. [21], in a cohort of 291 women, with recurrent miscarriage (RM) or RIF, who underwent 1304 assisted reproductive cycles, the authors evaluated the effect of the maternal KIR haplotype on the probability of a successful pregnancy, the risk of a stillbirth, and the number of live births after a single (SET) or double (DET) embryo transfer, depending on the oocytes provenance. The authors outlined the fact that pregnancy loss was more common in women with the KIR AA haplotype (22.8%), followed by those with the KIR AB haplotype (16.7%), and lowest in mothers with the KIR BB haplotype (11.1%; *p* = 0.03), following DET using the patient’s own oocytes. However, they could not confirm the same findings on the SET cohort of patients.

Another study by Alecsandru et al. [12], with a prospective design, evaluated the reproductive outcomes in 204 patients who underwent IVF according to maternal KIR genes expressed by uterine natural killer cells and paternal or oocyte donor HLA-C genes. The authors showed that compared to KIR AB (10.5% egg donation and 12.5% IVF) and KIR BB (6.7% egg donation and 0% IVF), KIR AA patients had a higher miscarriage rate following DETs (47.8% egg donation and 37.5% IVF). Moreover, they observed a significantly decreased LBR after DETs with oocyte donation in KIR AA patients (4.3%) compared with KIR AB (26.3%) or BB (46.7%), and a further decrease in the LBR as the fetal HLA-C2 load increased in KIR AA women. In this study, we did not have access to miscarriage tissue, nor did we have the possibility of testing the newborns for HLA-C genotyping. Our results showed that there was no statistically significant difference regarding the parental HLA-C genotypes between groups of patients with RIF and RPL. For this reason, the theoretical score proposed by Alecsandru et al. [12], that we chose to use for analysis, did not significantly differ among our groups.

On the other hand, a recent case–control study on 140 patients, investigated the association between KIR gene polymorphisms and unexplained recurrent pregnancy loss. The authors demonstrated that the KIR 2DL1, 2DL2, 2DL3, 2DL4, 2DS1, 2DS2, 2DS4, and 2DS5 polymorphisms, as well as Bx haplotypes, were associated with RPL [11]. In our study, the BB haplotype had equal prevalence in the RPL and RIF groups (66.7%), and no statistically significant difference between these groups could be determined in this regard. In our cohort of patients, the prevalence of AA haplotypes was higher in the RPL group, without reaching statistical significance (*p* = 0.51). Other studies found a significantly higher prevalence of KIR AA genotype in patients with RPL compared to a control population [22,23].

According to scant scientific evidence, the KIR genes (such as KIR2DL1 and KIR2DL2) that interact with HLA-C in trophoblast cells to transmit inhibitory signals for NK cell activity may be lacking in RPL females [24]. KIR2DL2-positive RPL females had lower HLA-C1 molecule concentrations than KIR2DL2-negative RPL females (*p* = 0.05) [25]. It is likely that insufficient ligands for inhibiting KIRs result in inadequate inhibition of maternal uNK cells to the trophoblast, causing RPL.

The recent literature outlined the immunological background for both RPL and RIF [7,8,26,27,28,29]. In this study we also evaluated if the personal history of autoimmune disorders varied in these cohorts of patients. The clinical data indicated that the personal history of SLE/APS, thrombophilia, and thrombosis were significantly more prevalent in the group of patients with recurrent pregnancy loss. A systematic review and meta-analysis indicated that pregnant women with hereditary thrombophilia had an increased risk of RPL, especially in the presence of G1691A mutation of the factor V Leiden (FVL) gene, the G20210A mutation of the prothrombin gene (PGM), and deficiency of protein S (PS) [30]. On the other hand, autoimmune disorders such as rheumatoid polyarthritis, autoimmune thyroiditis, and autoimmune thrombocytopenic purpura were not significantly associated with RPL or RIF, which was an aspect also confirmed by our study.

An interesting finding resulted from the analysis of paraclinical characteristics of the evaluated patients. Our findings outlined that the serum levels of ATPO were significantly higher in the RPL group compared to RIF group, and that the vitamin D serum levels were significantly lower in the RPL group compared to RIF group. In a case–control study by Yan et al., the authors compared the serum levels of vitamin D and the vitamin D receptors (VDR) expression in the chorionic villi and decidua, obtained during surgical evacuation of uterus of 40 women with RPL [31]. Their results indicated a reduced serum level of vitamin D, as well as reduced VDR expression in the chorionic villi and decidua of patients with RPL. Moreover, a recent literature review suggested that vitamin D supplementation might be beneficial for patients with RPL, although stronger evidence is needed before including this substance in the therapeutic scheme [32].

The limited size of our sample may imply a selection bias, which was one of our study’s shortcomings. Additionally, the heterogeneity of the clinical and paraclinical findings constituted a limitation in the relationship with the above-mentioned caveats. A more complete picture of the problem might be attained with a larger cohort of patients recruited from a variety of infertility centers. Additionally, the prevalence of KIR haplotypes varied in different regions of the world, and so, epidemiological studies on different populations of patients with RPL or RIF could outline a specific gene panel for each geographical region, and could properly assess the interaction effect between these gene panels and confounding factors.

The findings of this study suggest the need for a careful pregnancy follow-up for patients with RPL or RIF that underwent SET IVF and had an AA KIR haplotype. Clinicians should be aware of the adverse reproductive outcomes associated with this clinical context, and should provide comprehensive counselling to those patients, who were already confronted with pregnancy loss in various forms, and at different gestational ages. Additionally, even though supporting data is scarce, vitamin D supplementation could be beneficial for this category of patients, either as a part of preconception care or as soon as the pregnancy is confirmed.

## 5. Conclusions

Adverse reproductive outcomes are becoming more prevalent in recent times, especially in the context of an ascending trend of assisted reproductive techniques. It is important to clearly determine the association between clinical and paraclinical risk factors and adverse reproductive outcomes, as this would allow and improve the patient’s management.

This is the first prospective study that evaluated the influence of maternal KIR haplotypes on reproductive outcomes after single embryo transfer in IVF cycles in patients with recurrent pregnancy loss and implantation failure in Romania.

Our results indicated that patients with an AA haplotype had significantly higher chances of miscarriage if they underwent an IVF procedure, and it appeared that the same haplotype increased the chances of obtaining a pregnancy for patients who underwent an IVF procedure.

In this study, we found that the serum levels of vitamin D were significantly lower in the RPL group compared to the RIF group, suggesting that vitamin D supplementation might be beneficial for this category of patients, although further research is needed.

Further studies, on larger cohorts of patients with RPL or RIF from various geographical regions, could better characterize the KIR genes polymorphism and could potentially outline specific molecular targets for immunotherapies.

## Figures and Tables

**Table 1:** Clinical characteristics of the patients included in the main groups.

Patient’s Characteristics	Group 1 (RPL, n = 30)	Group 2 (RIF, n = 78)	*p* Value
Age, years (mean ± SD)	33.56 ± 3.95	33.92 ± 4.23	0.69
Medium (n/%)	Urban = 16 (53.3%)	Urban = 35 (44.9%)	
	Rural = 14 (46.7%)	Rural = 43 (55.1%)	0.43
Obesity (n/%)	Yes = 8 (26.7%)	Yes = 19 (24.4%)	0.80
Hypertension (n/%)	Yes = 3 (9%)	Yes = 7 (10%)	0.86
Renal disease (n/%)	Yes = 0 (0%)	Yes = 2 (2.6%)	0.37
Diabetes (n/%)	Yes = 1 (3.3%)	Yes = 2 (2.6%)	0.82
SLE/APS (n/%)	Yes = 7 (23.3%)	Yes = 4 (5.1 %)	0.005
Thrombophilia (n/%)	Yes = 6 (20%)	Yes = 2 (2.6%)	0.002
Thrombosis (n/%)	Yes = 5 (16.7%)	Yes = 2 (2.6%)	0.008
Endometriosis (n/%)	Yes = 7 (23.3%)	Yes = 22 (28.2%)	0.60
Autoimmune thyroiditis (n/%)	Yes = 6 (20%)	Yes = 8 (10.3%)	0.17
Rheumatoid arthritis (n/%)	Yes = 0 (0%)	Yes = 1 (1.3%)	0.55
Autoimmune thrombocytopenic purpura (n/%)	Yes = 0 (0%)	Yes = 1 (1.3%)	0.55

RPL—recurrent pregnancy loss; RIF: recurrent implantation failure; SD—standard deviation; APS—antiphospholipid syndrome; SLE—systemic lupus erythematosus.

**Table 2:** Paraclinical characteristics of the patients included in the main groups.

Patient’s Characteristics	Group 1 (RPL, n = 30)	Group 2 (RIF, n = 78)	*p* Value
AA haplotype (n/%)	Yes = 10 (33.3%)	Yes = 21 (26.9%)	0.51
AB haplotype (n/%)	Yes = 0 (0%)	Yes = 4 (5.71%)	0.20
BB haplotype (n/%)	Yes = 20 (66.7%)	Yes = 53 (66.7%)	0.99
Maternal HLA- C1/C2 (n/%)	Yes = 14 (46.7%)	Yes = 33 (42.3%)	0.68
Maternal HLA- C2/C2 (n/%)	Yes = 7 (23.3%)	Yes = 14 (17.9%)	0.52
Maternal HLA- C1/C1 (n/%)	Yes = 31 (39.7%)	Yes = 14 (17.9%)	0.20
Partner’s HLA- C1/C2 (n/%)	Yes = 18 (60%)	Yes = 43 (55.1%)	0.64
Partner’s HLA- C2/C2 (n/%)	Yes = 2 (6.7%)	Yes = 13 (16.7%)	0.17
Partner’s HLA- C1/C1 (n/%)	Yes = 10 (33.3%)	Yes = 22 (28.2%)	0.60
Score (mean ± SD)	0.73 ± 0.58	0.85 ± 0.67	0.37
TSH, μUI/mL, (mean ± SD)	2.10 ± 1.07	1.88 ± 0.98	0.33
fT4, pmol/L, (mean ± SD)	11.48 ± 4.18	12.88 ± 3.77	0.14
ATPO, UI/mL, (mean ± SD)	140.32 ± 378.48	50.89 ± 156.99	0.005
Vitamin D, ng/mL, (mean ± SD)	32.76 ± 21.98	39.91 ± 20.13	0.006
AMH, ng/mL, (mean ± SD)	2.98 ± 2.18	2.89 ± 1.99	0.41

RPL—recurrent pregnancy loss; RIF: recurrent implantation failure; SD—standard deviation; TSH—thyroid stimulating hormone; fT4—free thyroxine; ATPO—anti-thyroid peroxidase antibodies; AMH—anti-müllerian hormone.

**Table 3:** Comparison of biohumoral parameters for the patients included in the analyzed subgroups.

		Sum of Squares	Mean Square	F Score	*p* Value
Score	Between Groups	0.427	0.213	0.496	0.611
TSH	Between Groups	1.371	0.685	0.667	0.516
fT4	Between Groups	4.656	2.328	0.149	0.861
ATPO	Between Groups	22,904.490	11,452.245	0.201	0.818
AMH	Between Groups	3.024	1.512	0.360	0.698
Vitamin D	Between Groups	250.316	125.158	0.473	0.625

TSH—thyroid stimulating hormone; fT4—free thyroxine; ATPO—anti-thyroid peroxidase antibodies; AMH—anti-müllerian hormone.

**Table 4:** Comparison of reproductive outcomes between patients with various haplotypes.

Reproductive Outcome	AA	AB	BB	*p* Value
PR	25 (80.6%)	4 (80%)	68 (94.4%)	0.08
MR	7 (22.6%)	1 (20%)	5 (6.9%)	0.018
LBR	18 (58.1%)	3 (60%)	63 (87.5%)	0.003

PR—pregnancy rate; MR: miscarriage rate; LBR—live birth rate.

**Table 5:** Comparison of reproductive depending on the mode of achieving a pregnancy for the patients included in the analyzed subgroups.

**Kir Haplotype**	**MR (Odds ratio, 95% CI lower limit–upper limit)**						
	**LBR**						
	**S**			**IVF**			***p*** **Value**
AA	2.36 (0.84–8.38)			4.15 (1.39–6.50)			0.032
AB	0.70 (0.46–1.06)			1.01 (0.98–1.05)			0.314
BB	0.43 (0.16–1.14)			0.98 (0.96–1.00)			0.051
**Kir Haplotype**	**PR (Odds ratio, 95% CI lower limit–upper limit)**						
	**S**			**IVF**			***p* Value**
AA	1.04 (0.97–1.11)			2.57 (0.85–6.75)			0.023
AB	1.01 (0.98–1.05)			1.03 (0.97–1.08			0.251
BB	0.98 (0.96–1.00)			1.15 (0.74–1.78)			0.518
**Kir Haplotype**	**LBR (Odds ratio, 95% CI lower limit–upper limit)**						
	**S**			**IVF**			***p* Value**
AA	0.51	0.18	1.44	0.84	0.64	1.09	0.174
AB	0.75	0.12	4.59	0.94	0.30	2.92	0.759
BB	1.12	0.94	1.34	1.62	0.27	6.70	0.189

PR—pregnancy rate; MR: miscarriage rate; LBR—live birth rate; S—spontaneous pregnancy; IVF—pregnancy obtained by in vitro fertilization.

## Data Availability

The data presented in this study are available on request from the corresponding author. The data are not publicly available due to local policies.
